# Continuing Professional Development ‐ Radiation Therapy

**DOI:** 10.1002/jmrs.770

**Published:** 2024-02-24

Maximise your CPD by reading the following selected article and answer the five questions. Please remember to self‐claim your CPD and retain your supporting evidence. Answers will be available via the QR code and online at www.asmirt.org/news‐and‐publications/jmrs, as well as published in JMRS – Volume 71, Issue 4, December 2024.

## Radiation Therapy – Original Article

### Travelling overseas for proton beam therapy: A retrospective interview study

Skelton K, Gorayski P, Tee H, Anderson N, Le H. (2024). *J Med Radiat Sci*. https://doi.org/10.1002/jmrs.721

1. What is the primary advantage of proton beam therapy (PBT) over conventional photon treatment mentioned in the article?
  - PBT is more cost‐effective
  - PBT uses high‐energy x‐ray beams
  - PBT has a longer treatment duration
  - PBT delivers radiation directly at the tumour with sparing of normal tissues
2. How do families in Australia currently access PBT treatment?
  - Through a local government program
  - By self‐funding or applying to the Medical Treatment Overseas Program (MTOP)
  - Only through participation in clinical trials
  - Exclusively through the Royal Adelaide Hospital (RAH)
3. What does the Australian Medical Treatment Overseas Program (MTOP) provide financial assistance for?
  - Only the cost of radiation therapy
  - Treatment, accommodation and airfares for the patient and one support person
  - Only airfares for the patient
  - All medical expenses, including surgery and medications
4. In the study, what was identified as a significant gap in the literature regarding patients travelling for cancer treatment?
  - Perceived experiences of patients and families travelling overseas for PBT
  - Lack of information about treatment centres
  - Economic burden for urban Australian patients
  - Experiences of patients travelling for surgery
5. According to the research in the article, what challenges do paediatric and adolescent patients, along with their families, face when given a cancer diagnosis?
  - Lack of treatment options
  - Difficulty in accessing conventional radiation therapy
  - Need for a supportive care model of practice
  - Limited financial assistance from the MTOP

### Recommended further reading:

1. Kinahan KE, Kircher S, Altman J, et al. Promoting the shared‐care model for adolescent and young adults with cancer: optimizing referrals and care coordination with primary care providers. *J Natl Compr Canc Netw* 2017; 15(1): 38–44. https://doi.org/10.6004/jnccn.2017.0005
2. Knibbs V, Manley S. Being away from home for cancer treatment: a qualitative study of patient experience and supportive care needs during radiation therapy. *J Med Radiat Sci* 2022; 69(3): 336–47. https://doi.org/10.1002/jmrs.578

## Answers

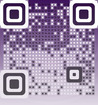

Scan this QR code to find the answers, or visit www.asmirt.org/news‐and‐publications/jmrs

